# Treatments and costs for recurrent and/or metastatic squamous cell carcinoma of the head and neck in the Netherlands

**DOI:** 10.1007/s00405-015-3495-y

**Published:** 2015-01-21

**Authors:** Naomi van der Linden, Jan Buter, Chris P. Pescott, Roy I. Lalisang, Jan Paul de Boer, Alexander de Graeff, Carla M. L. van Herpen, Robert J. Baatenburg de Jong, Carin A. Uyl-de Groot

**Affiliations:** 1 Institute for Medical Technology Assessment, Erasmus University Rotterdam, Woudestein location (J5-51), P.O. Box 1738, 3000 DR Rotterdam, The Netherlands; 2 VU University Medical Center, De Boelelaan 1117, 1081 HV Amsterdam, The Netherlands; 3 Division of Medical Oncology, Department of Internal Medicine, GROW, School of Oncology and Developmental Biology, Maastricht University Medical Center, P.O. Box 616, 6200 MD Maastricht, The Netherlands; 4 Netherlands Cancer Institute/Antoni van Leeuwenhoek, P.O. Box 90203, 1006 BE Amsterdam, The Netherlands; 5 University Medical Center Utrecht, P.O. Box 85500, 3508 GA Utrecht, The Netherlands; 6 Radboud University Medical Center, P.O. Box 9101, 6500 HB Nijmegen, The Netherlands; 7 Erasmus Medical Center, P.O. Box 2040, Rotterdam, 3000 CA The Netherlands; 8 Institute for Medical Technology Assessment, Erasmus University Rotterdam, P.O. Box 1738, 3000 DR Rotterdam, The Netherlands; 9 Merck KGaA, Frankfurter Str. 250, F135/101, 64293 Darmstadt, Germany

**Keywords:** Carcinoma, squamous cell of head and neck, Drug therapy, Costs and cost analysis

## Abstract

For patients with recurrent and/or metastatic squamous cell carcinoma of the head and neck (R/M SCCHN), chemotherapy can prolong life and alleviate symptoms. However, expected gains may be small, not necessarily outweighing considerable toxicity and high costs. Treatment choice is to a large extent dependent on preferences of doctors and patients and data on these choices are scarce. The purpose of this study is to obtain real-world information on palliative systemic treatment and costs of R/M SCCHN in the Netherlands. In six Dutch head and neck treatment centers, data were collected on patient and tumor characteristics, treatment patterns, disease progression, survival, adverse events, and resource use for R/M SCCHN, between 2006 and 2013. 125 (14 %) out of 893 R/M SCCHN patients received palliative, non-trial first-line systemic treatment, mainly platinum + 5FU + cetuximab (32 %), other platinum-based combination therapy (13 %), methotrexate monotherapy (27 %) and capecitabine monotherapy (14 %). Median progression-free survival and overall survival were 3.4 and 6.0 months, respectively. 34 (27 %) patients experienced severe adverse events. Mean total hospital costs ranged from €10,075 (±€9,891) (methotrexate monotherapy) to €39,459 (±€21,149) (platinum + 5FU + cetuximab). Primary cost drivers were hospital stays and anticancer drug treatments. Major health care utilization and costs are involved in systemically treating R/M SCCHN patients with a limited survival.

## Introduction

In the Netherlands, 2,970 new cases of head and neck cancer were diagnosed in 2011, approximately 1 per 6,000 inhabitants [1]. In up to 90 % of cases, this concerns squamous cell carcinoma (SCCHN) [2]. Approximately 17 % of SCCHN patients develop local tumor recurrence, 10 % of patients develop regional tumor recurrence and 11 % progress to distant metastatic disease [3]. Distant metastases are present at initial diagnosis in 1.8 % of patients [3]. Median survival for patients with recurrent and/or metastatic SCCHN (R/M SCCHN) is 6–9 months [4].

For some patients with loco-regional tumor recurrence, surgery or radiotherapy may still cure the disease [5]. For patients with non-curable loco-regional tumor recurrence and patients with distant metastasis, palliation may be offered by surgery, radiotherapy, photodynamic therapy (PDT), or systemic treatment.

Radiotherapy may be used for loco-regional recurrent tumors for radiation naïve patients or when re-irradiation is possible, typically with curative intent. Radiotherapy is also the mainstay therapy to treat symptomatic bone metastases. Systemic treatment may be used for the palliative treatment of loco-regional recurrent disease and/or distantly metastasized tumors. However, this treatment is only considered in case of good performance status and symptoms related to tumor growth. The primary aim of palliative chemotherapy is to alleviate symptoms [6–8].

Active pharmaceutical agents registered for palliative treatment in R/M SCCHN include the platinum compounds (cisplatin and carboplatin), 5-fluorouracil (5FU), methotrexate, taxanes, bleomycin, and the monoclonal antibody cetuximab [9]. They can be used as monotherapy or in various combination regimens. No new compounds have been identified in the past 5 years that demonstrate clinical benefit in late stage clinical trials.

Historically, the usual first-line treatment for incurable SCCHN has been combination chemotherapy with cisplatin and 5FU. For clinically fit patients (performance score 0–1), international guidelines [9, 10] advise treatment with platinum plus 5FU and cetuximab. Cetuximab, an EGFR inhibitor added to platinum-5FU, increased overall survival (median 10.1 vs. 7.4 months) and progression-free survival (median 5.6 vs. 3.3 months) in a randomized controlled phase III trial [11]. In November 2009, the scientific committee (CieBOM) of the Dutch Association for Medical Oncology (NVMO) considered addition of cetuximab to platinum-5FU to provide added therapeutic benefit for clinically fit patients with R/M SCCHN [12].

Treatment with single agents may be offered to patients who may not tolerate combination chemotherapy. For these patients, Dutch guidelines recommend methotrexate monotherapy. Although response percentages with methotrexate are lower than with platinum-5FU, overall survival is similar [13].

Due to possible side effects and limited clinical benefit of palliative systemic treatment in R/M SCCHN, treatment choice is, to a large extent dependent on individual preferences of doctors and their patients. In the Netherlands, a lack of data exists on daily practice treatment patterns, survival, adverse events and costs associated with management of R/M SCCHN. The aim of this study is to provide insight into these outcome measures.

## Methods

### Data collection

More than 90 % of SCCHN patients are treated in one of the head and neck treatment center [14], making head and neck cancer care a highly centralized field of medicine in the Netherlands. A retrospective, observational study was conducted in six of a total of eight Dutch head and neck treatment centers. Patients were identified from hospital and pharmacy databases.

Medical charts were reviewed for patients diagnosed with recurrent and/or metastatic (M+) squamous cell carcinoma of the head and neck (ICD-O C01–C14 and C30–C32) between January 1, 2006 and July 3, 2013. Recurrence was defined as occurring within 2 cm of the original tumor or lymph node site and within 5 years after primary treatment of the initial, usually locally advanced, tumor. Data on all local and systemic treatments were recorded on case report forms. For all study patients with at least one line of palliative, non-trial systemic treatment, additional patient and tumor characteristics, treatment details, resource use and clinical outcomes were collected. Information on treatment history was collected as well, but not used for selection purposes. Patients who only received systemic treatment in a clinical trial (*n* = 20), were excluded from this extensive data collection since we aimed to present real-world, daily practice treatment patterns and outcomes. For patients treated in trials, management and therefore resource use are usually guided by the trial protocol and, therefore, not representative of daily practice.

Comorbidity was determined from medical records, measured at baseline, using the updated Charlson comorbidity index. This index is valid for head and neck cancer patients and predicts the 1-year in-hospital mortality based on comorbidity [15, 16].

### Clinical outcomes

Overall survival (OS) was defined as the duration between date of treatment start (for the first palliative, systemic, non-trial treatment) and date of death as registered in the hospital record. For none of the patients a cause of death other than head and neck cancer was registered. Progression-free survival (PFS) was defined as the time from treatment start to disease progression, defined as: (1) clinical or radiological progression of recurrent tumor and/or distant metastases; (2) start of new treatment (with the exception of treatment change due to toxicity); or (3) death, whichever occurred first. A second primary tumor was not classified as disease progression.

Adverse events (AEs) reported in the patient chart and graded by a physician were recorded using the Common Terminology Criteria for Adverse Events (case report form based on CTC version 4.03). Adverse events for which no grade was provided were recorded as severe adverse events if they resulted in hospital admission or dose reduction, postponement or change of treatment. No AE information was derived from laboratory values or administered treatments.

### Economic outcomes

Resource use included in-patient hospital days, day-care hospital admissions, outpatient visits, drug usage, radiotherapy, surgery and other invasive procedures, laboratory diagnostics, imaging and pathology. Drug use other than anti-cancer drugs, including treatments for adverse events, was determined in a sub-selection of patients (*n* = 49), for reasons of feasibility. Mean per patient treatment costs were calculated combining resource use and unit costs, derived from literature [17, 18] or official tariff lists. Treatment costs were calculated from start of the respective treatment onwards and include all subsequent resource use. Costs are reported from the head and neck cancer center perspective, in Euros. Unit costs are from 2013 or were inflated to reflect the 2013 price level.

### Analyses

Descriptive analyses were performed in IBM SPSS Statistics 21. The Kaplan–Meier method was used for survival estimates.

## Results

### Treatment patterns

**Fig. 1:**
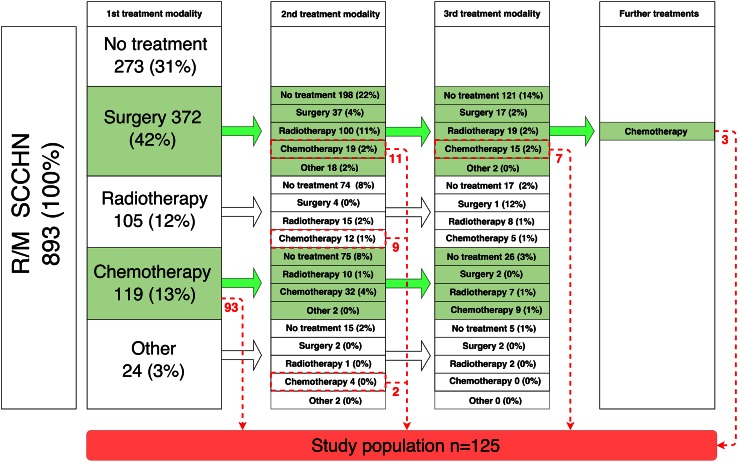
Treatment patterns for R/M SCCHN patients. The *red numbers* (color version online) represent eligible patients, therefore, included in the study. For the sake of readability, treatments after the third line were not further specified

893 patients diagnosed with R/M SCCHN were identified (Fig. 1), 20 of whom received systemic trial treatment only. If patients received trial treatment at one point in time but non-trial systemic treatment at another point in time, these patients were included from start of the non-trial treatment onwards (costs for trial treatment are set to €0 from a hospital perspective). 273 patients received no antitumor treatment at all. 125 patients received at least one line of palliative, non-trial systemic treatment and were included in the study. Of these 125 study patients, 7 patients had metastasized SCCHN at primary diagnosis and 118 patients had R/M SCCHN after primary treatment. 93 study patients received non-trial systemic treatment as first treatment after diagnosis of R/M SCCHN and 32 study patients as second, third or fourth treatment.

### Treatment characteristics

Multiple treatment modalities were administered (Fig. 1). The most common first-line systemic treatment choices (Table 1) were platinum + 5FU + cetuximab (*n* = 40, 32 %), other platinum-based combination therapies (*n* = 16, 13 %), methotrexate monotherapy (*n* = 34, 27 %) and capecitabine monotherapy (*n* = 18, 14 %). An example of an “other” first-line drug therapy was platinum monotherapy (*n* = 9). Patients treated with first-line platinum-based combination therapy without cetuximab received platinum + fluorouracil (*n* = 6), cisplatin + gemcitabine (*n* = 4), platinum + capecitabine (*n* = 2), and other platinum combination regimens (*n* = 4).

**Table 1:** Drug treatment in daily practice

Treatment	First systemic treatment line (*n* = 125)	Second systemic treatment line (*n* = 39)
Platinum + 5FU + cetuximab	40 (32 %)	4 (10 %)
Other platinum-based combination therapy	16 (13 %)	1 (3 %)
Methotrexate monotherapy	34 (27 %)	15 (38 %)
Capecitabine monotherapy	18 (14 %)	7 (18 %)
Other	17 (14 %)	12 (31 %)

The percentage of patients treated with platinum + 5FU + cetuximab has increased steeply since 2010 (data not presented), following a positive decision on reimbursement. In patients receiving platinum + 5FU + cetuximab, 40 patients (32 %) received this combination in first line, 4 (10 %) in second line, and 0 (0 %) in subsequent treatment lines. Other platinum-based combination therapies were administered to 16 patients (13 %) as first-line therapy and to 1 (3 %) in second line. This regimen was administered as subsequent treatment to 1 patient (14 %).

In the second systemic treatment line, methotrexate monotherapy was the most frequently prescribed drug regimen.

### Patient and tumor characteristics

Patient and tumor characteristics are depicted in Table 2. 74 % of patients were male and the median age was 60. Unfortunately, performance status was not routinely registered in all medical charts.

**Table 2:** Patient and tumor characteristics, stratified by first systemic treatment line group

	Total (*n* = 125)	Platinum + 5FU + cetuximab (*n* = 40)	Platinum-based combination therapy (*n* = 16)	Methotrexate monotherapy (*n* = 34)	Capecitabine monotherapy (*n* = 18)	Other (*n* = 17)
Sex, *n* (%)						
Male	92 (74)	28 (70)	9 (56)	27 (79)	13 (72)	15 (88)
Median age	60	58	57	62	62	60
Age (years), *n* (%)						
<65	90 (72)	33 (83)	14 (88)	21 (62)	11 (61)	11 (65)
≥65	35 (28)	7 (18)	2 (13)	13 (38)	7 (39)	6 (35)
Primary tumor site, *n* (%)						
Oropharynx	38 (30)	10 (25)	6 (38)	11 (32)	6 (33)	5 (29)
Hypopharynx	21 (17)	5 (13)	2 (13)	5 (15)	1 (6)	8 (47)
Larynx	17 (14)	5 (13)	1 (6)	9 (27)	2 (11)	0 (0)
Oral cavity	34 (27)	16 (40)	2 (13)	9 (27)	5 (28)	2 (12)
Nasopharynx	10 (8)	2 (5)	5 (31)	0 (0)	1 (6)	2 (12)
Other	5 (4)	2 (5)	0 (0)	0 (0)	3 (17)	0 (0)
Extent of disease, *n* (%)						
Loco-regionally recurrent	58 (47)	20 (50)	6 (38)	18 (53)	9 (50)	5 (29)
Metastatic with or without loco-regional recurrence	67 (54)	20 (50)	10 (63)	16 (47)	9 (50)	12 (71)
Location of distant metastases, *n* (%)						
Bone(s)	18 (14)	7 (18)	5 (31)	2 (6)	2 (11)	2 (12)
Lung	54 (43)	16 (40)	7 (44)	15 (44)	6 (33)	10 (59)
Liver	15 (12)	5 (13)	5 (31)	1 (3)	1 (6)	3 (18)
Lymph nodes	24 (19)	10 (25)	3 (19)	6 (18)	2 (11)	3 (18)
Skin	13 (10)	8 (20)	1 (6)	1 (3)	2 (11)	1 (6)
Other	10 (8)	4 (10)	1 (6)	3 (9)	2 (11)	0 (0)
Comorbidity, *n* (%)						
0	110 (88)	35 (88)	14 (88)	31 (91)	16 (89)	14 (82)
1	11 (9)	4 (10)	2 (13)	3 (9)	1 (6)	1 (6)
>1	4 (3)	1 (3)	0 (0)	0 (0)	1 (6)	2 (12)
Previous treatments^a^, *n* (%)						
No previous treatments	11 (9)	6 (15)	2 (13)	2 (6)	0 (0)	1 (6)
Surgery(s) and or radiotherapy(s) only	73 (58)	25 (63)	8 (50)	22 (65)	8 (44)	10 (59)
Chemoradiation	37 (30)	9 (23)	6 (38)	9 (27)	8 (44)	5 (29)
Chemotherapy	3 (2)	1 (3)	0 (0)	0 (0)	1 (6)	1 (6)
Any cetuximab	3 (2)	0 (0)	0 (0)	1 (3)	1 (6)	1 (6)
Months between initial diagnosis SCCHN and diagnosis R/M SCCHN (mean, SD)	15.6, 17.5	14.2, 16.5	20.6, 25.1	18.8, 19.9	13.2, 7.0	17.3, 14.0
Months between diagnosis R/M SCCHN and start first palliative systemic therapy (mean, SD)	3.9, 6.1	4.1, 6.9	3.9, 7.6	4.0, 4.7	4.3, 8.0	2.6, 2.2

^a^Antitumor treatments for SCCHN before diagnosis of recurrence and/or metastasis. Treatment history was not a selection criterion for this study

### Survival measures

Table 3 shows PFS and OS per treatment group, from treatment start onwards, without correction for baseline characteristics. Median PFS and OS for the cohort studied were 3.4 and 6.0 months, respectively. Due to heterogeneity, possibilities for matching on baseline characteristics were limited and did not solve the issue of confounding by indication. Therefore, survival estimates should be interpreted as descriptive of the respective treatment groups rather than measures of treatment effect. Wide, overlapping confidence intervals reflect non-significance of the survival differences, due to small size of the treatment groups.

**Table 3:** Overall survival and progression-free survival per treatment group

First systemic treatment line	Overall survival, median (95 % CI)	Progression-free survival, median (95 % CI)
Platinum + 5FU + cetuximab (*n* = 40)	6.7 (4.4–8.9)	4.8 (3.2–6.4)
Other platinum-based combination therapy (*n* = 16)	10.5 (5.8–15.1)	4.0 (3.5–4.4)
Methotrexate monotherapy (*n* = 34)	4.8 (3.5–6.1)	3.1 (1.9–4.3)
Capecitabine monotherapy (*n* = 18)	3.7 (1.4–5.9)	1.7 (1.5–1.9)
Other (*n* = 17)	5.7 (1.2–10.3)	1.6 (0.3–2.9)
All (*n* = 125)	6.0 (4.2–7.8)	3.4 (2.3–4.5)

Due to heterogeneity, possibilities for matching on baseline characteristics were limited and did not solve the issue of confounding by indication. Therefore, survival estimates should be interpreted as descriptive of the respective treatment groups rather than measures of treatment effect

### Adverse events

In the initial palliative treatment line, 34 patients (27 %) experienced severe adverse events, defined as any adverse events with registered record of: CTC AE grade ≥3, treatment dose reduction(s), postponement or change of treatment, and/or hospital admission. 21 hospital stays (4 % of total hospital stays) resulted from AEs, for a total of 16 patients (13 %). Median duration of these hospital stays was 8 days. Severe adverse events were observed more often in patients receiving combination therapy than methotrexate or capecitabine monotherapy (Table 4).

**Table 4:** Adverse events

First systemic treatment regimen	Severe adverse events, *n* (%)	Reported severe adverse events CTC AE grade ≥III, patient was hospitalized, and/or treatment was adapted for toxicity reasons	Reported non-severe adverse events CTC AE grade I and grade II^a^
Cisplatinum +5FU + cetuximab (*n* = 40)	19 (48 %)	Anorexia, cardiac toxicity, ear and labyrinth disorder, febrile neutropenia, hand–foot syndrome, nausea, oral mucositis, thrombocytopenia, pneumonia, renal toxicity and skin and subcutaneous tissue disorders	Acneiform rash, constipation, diarrhea, dehydration, dry skin, fatigue, erythema multiforme, hand–foot syndrome, hypokalemia, mucositis, nausea, other skin and subcutaneous tissue disorders, ototoxicity, pain, papulopustular rash, pruritus, renal disorders and vomiting
Other platinum-based combination therapy (*n* = 16)	5 (31 %)	Diarrhea, febrile neutropenia, renal disorder and vomiting	Anorexia, dysphagia, dry skin, fatigue, hand–foot syndrome, leukopenia, nausea, pneumonia and vomiting
Methotrexate monotherapy (*n* = 34)	5 (15 %)	Liver toxicity, malaise, neutropenia, and oral mucositis	Dysphagia, pneumonia, pain and fatigue
Capecitabine monotherapy (*n* = 18)	0 (0 %)	None reported	None reported
Other (*n* = 17)	5 (29 %)	Renal disorders, cardiac disorder, fatigue and constipation	Alopecia and nausea

^a^Although these adverse events were only recorded if their severity had been assessed by a physician and reported in the patient file, we could not make a clear distinction between grade I and grade II adverse events due to non-specificity in reporting habits (reading, for example, “headache grade I/II” or “low-grade headache”)

### Costs

Table 5 presents mean costs per treatment group and cost category. Mean total costs per patient were €24,211 (±€22,432), ranging from €10,075 (±€9,891) (methotrexate monotherapy) to €39,459 (±€21,149) (platinum + 5FU + cetuximab). Primary cost drivers are hospital stays and drug costs.

**Table 5:** Costs in 2013 € per treatment group

	Cisplatin + 5FU + cetuximab (*n* = 40), mean (SD)	Other platinum-based combination therapy (*n* = 16), mean (SD)	Methotrexate monotherapy (*n* = 34), mean (SD)	Capecitabine monotherapy (*n* = 18), mean (SD)	Other (*n* = 17), mean (SD)	All (*n* = 125), mean (SD)
In-patient hospital days (€)	16,564 (11,396)	18,823 (13,267)	5,752 (7,563)	2,834 (3,874)	8,837 (8,657)	10,884 (11,173)
Intensive care unit days (€)	544 (2,572)	0 (0)	58 (339)	0.00 (0.00)	0.00 (0.00)	190 (1,473)
Day-care treatment (€)	1,977 (2,601)	2,143 (3,271)	709 (1,039)	1,013 (2,272)	836 (1,170)	1,359 (2,229)
Outpatient visits (€)	2,186 (2,153)	4,366 (3,793)	1,260 (2,012)	1,567 (2,282)	655 (972)	1,916 (2,502)
Anticancer drug treatment^a^ (€)	14,938 (9,085)	5,770 (7,375)	731 (1,800)	3,232 (7,468)	4,368 (7,569)	6,777 (9,094)
Concomitant medication^b^ (€)	482 (779) *n* = 12	346 (462) *n* = 4	194 (279) *n* = 14	233 (355) *n* = 8	256 (361) *n* = 11	297 (481) *n* = 49
Palliative anticancer surgery, photodynamic therapy, radiotherapy (€)	656 (1,442)	4,307 (6,164)	551 (1,347)	624 (1,427)	1,102 (1,760)	1,151 (2,814)
Laboratory (€)	878 (475)	1,274 (1,192)	481 (452)	430 (611)	642 (678)	724 (696)
Imaging, nuclear medicine, procedure (€)	1,686 (1,843)	1,849 (1,993)	511 (595)	863 (2,516)	1,039 (1,029)	1,181 (1,711)
Pathology (€)	31 (58)	52 (115)	21 (44)	21 (91)	26 (64)	29 (70)
Total^c^ (€)	39,459 (21,149)	38,584 (26,065)	10,075 (9,891)	10,585 (14,544)	17,506 (16,634)	24,211 (22,431)
Duration of period (months)	7.3 (6.3)	19.2 (17.9)	6.5 (7.8)	6.0 (5.9)	7.8 (6.4)	8.5 (9.8)
Costs/month	7,537 (4,105)	3,520 (2,692)	3,013 (2,234)	2,779 (4,749)	2,525 (2,038)	4,214 (4,196)

^a^Including drug spillage

^b^As determined in a side study among a random sample of 49 patients from VUmc and UMCU, since these hospitals kept electronic medication records with enough detail to determine costs

^c^Excluding concomitant medication costs, since these were determined for only 39.2 % of the patient sample

## Discussion

Relatively few (14 %) patients in the Netherlands with R/M SCCHN received palliative systemic treatment. Patient and treatment heterogeneity as well as small sample size prevented us from statistically comparing treatment costs and outcomes. The most frequently prescribed first-line drug regimen consists of cisplatin + 5FU + cetuximab, followed by methotrexate monotherapy. In the second systemic treatment line, methotrexate monotherapy is the most frequently prescribed drug regimen. Treatment with single agents is associated with fewer adverse events than combination treatments. The choice of treatment is hospital dependent (stratified data not presented for confidentiality reasons).

A multi-country survey of 256 head and neck specialists in France, Germany, Italy and Spain showed that 72 % of R/M SCCHN patients were treated with first-line combination therapy: 65 % of these patients were treated with cetuximab containing regimens and 35 % with other platinum-based combination chemotherapy. Combination treatment with cetuximab is a common first-line choice in these countries (data published as abstract only) [19]. In the Netherlands, head and neck cancer specialists seem to take a more conservative approach with respect to prescribing chemotherapy in general and platinum + 5FU + cetuximab in particular (32 % of all palliative, first line, non-trial, systemic regimens). However, the difference could be explained by different study designs, recall bias and possibly a preselected patient population of the head and neck specialists in the multi-country survey. It is likely that survey results provide less reliable information on treatment allocation than medical chart review for all diagnosed R/M SCCHN patients.

For the study population as a whole, median overall survival from diagnosis was 6.0 months. Patients treated with combination platinum regimens other than platinum + 5FU +cetuximab live longer, possibly due to their lower age and a higher proportion of tumors that are relatively sensitive to treatment, such as nasopharyngeal carcinomas. Nasopharyngeal carcinomas are a distinct subgroup known to respond differently to treatment than SCCHN in other localizations. They constitute a relatively favorable prognostic group [20].

Survival of 95 % CIs of patients treated with platinum + 5FU + cetuximab in Dutch daily practice (median OS 6.7 months, 95 % CI 4.4–8.9, median PFS 4.8, 95 % CI 3.2–6.4) overlap with those from the EXTREME trial [11] (median OS 10.1 months, 95 % CI 8.6–11.2, median PFS 5.6, 95 % CI 5.0–6.0) and a retrospective, observational study from Portugal [21] (median OS 11 months, 95 % CI 8.7–13.3, median PFS 8, 95 % CI 6.1–9.9).

The data presented are the only published evidence on the costs of systemically treated R/M SCCHN in the Netherlands. Hospital stays and chemotherapeutics are the main cost drivers. We report mean costs of management of systemically treated R/M SCCHN of € 24,211. These costs are considerable, yet not as high as published end-of-life healthcare consumption for various cancers in a US study population (inpatient and outpatient costs $70,956, in 2009 USD) [22]. For the Netherlands, mean costs of late stage cancer management have not been explored in great detail.

Costs incurred for cancer care do not automatically result in better outcomes [23]. Policy makers, oncologists and public media increasingly express the need to curtail the rise in costs of cancer care. Suggested changes include limiting the use of chemotherapy combination regimens for metastatic cancers and limiting chemotherapy on the basis of performance status [24]. Even disregarding the costs, extensive use of chemotherapy at the end of life can be an important signal of poor quality care [25]. Our study shows relatively few R/M SCCHN patients to receive systemic palliative treatment, which might reflect careful patient selection due to the small expected gains of such treatments, considerable toxicity and high costs.

Still the presented cost estimates raise the question about the value for money that is achieved. Very little is known about this for the R/M SCCHN patient population. There is relatively little high-quality research in these patients, possibly due to rarity of the disease in western countries, heterogeneity within the patient population (amongst others in tumor localization), lack of new treatment compounds, and difficulties associated with quality of life measurements in end stage cancer patients. To our knowledge, no pharmacoeconomic studies have been published about systemic R/M SCCHN treatments except for cost-effectiveness studies regarding platinum + 5FU + cetuximab versus platinum + 5FU [26, 27].

Analysis of the cost-effectiveness of systemic treatments in daily practice requires information about (changes in) health-related quality of life and a large enough patient population to compare treatment strategies while correcting for confounding by indication. Preferably these data should be collected within a population-based patient registry, including all newly diagnosed patients with head and neck SCC in the Netherlands. Such a register has the potential to boost the quality of head and neck cancer research and has a reasonable feasibility in the Netherlands due to the centralized nature of head and neck cancer care. However, several challenges exist regarding patient identification as well as patient follow-up in the terminal phase.

### Limitations of the study

Squamous cell carcinoma of the head and neck patients form a relatively small and heterogeneous population. This limited possibilities to correct for confounding by indication. As a result the effect of treatment choice on outcomes could not be assessed and only descriptive results were presented.

Furthermore, the level of detail in medical records varied greatly. This prevented uniform capture of several variables, such as performance status and adverse events. For example, the lack of adverse events seen in patients receiving capecitabine monotherapy could be due to a less intensive follow-up since this treatment is self-administered at home. The lack of certain anticipated adverse events, such as hypomagnesaemia with the platinum-based treatments, results from the data managers recording AEs only when explicitly reported by clinicians, without, for example, consulting laboratory values themselves.

Notably, our research was conducted in patients identified through hospital records and focused on treatment in a specialized head and neck center setting. Some 90 % of SCCHN patients in the Netherlands visit these head and neck centers [14]. However, patients who do not seek specialized medical care were not included in this study. Therefore, the proportion of patients not receiving systemic therapy is likely to be underestimated. Furthermore, two out of eight head and neck centers did not participate in the study and might have had different treatment patterns. Also, hospital and pharmacy databases can be incomplete, especially when patients had only few hospital contacts.

Resource consumption of interventions offered outside the study hospital, i.e. for patients referred to other (outpatient) clinics for drug administration, was not recorded. Therefore, presented cost estimates reflect the costs incurred within the head and neck treatment centers. Cost utility of treatments for R/M SCCHN could not be assessed due to a lack of comprehensive outcomes reporting, specifically on quality of life.

## Conclusion

For systemically treated patients with R/M SCCHN, health care utilization and associated costs are considerable, while the survival is limited.

## Abbreviations

5FU: 5-Fluorouracil
AE: Adverse event
CI: Confidence interval
OS: Overall survival
PDT: Photodynamic therapy
PFS: Progression-free survival
R/M SCCHN: Recurrent and/or metastatic squamous cell carcinoma of the head and neck

## References

[1] Netherlands Cancer Registry (2013) Kerncijfers. http://www.cijfersoverkanker.nl (Accessed Jan 2014)

[2] Haines G (2013) Pathology of head and neck neoplasms. In: UpToDate

[3] van der Schroeff MP, Steyerberg EW, Wieringa MH, Langeveld TP, Molenaar J, Baatenburg de Jong RJ (2012). Prognosis: a variable parameter: dynamic prognostic modeling in head and neck squamous cell carcinoma. Head Neck.

[4] Brockstein B, Vokes E, Basow D (2013). Treatment of metastatic and recurrent head and neck cancer. UpToDate.

[5] Ledeboer QC, van der Schroeff MP, Pruyn JF, de Boer MF, Baatenburg de Jong RJ, van der Velden LA (2011). Survival of patients with palliative head and neck cancer. Head Neck.

[6] Nederlandse Werkgroep Hoofd-Hals Tumoren (2010) Landelijke richtlijn hypofarynxcarcinoom, versie 2.0. http://www.oncoline.nl/hypofarynxcarcinoom (Accessed Jan 2014)

[7] Nederlandse Werkgroep Hoofd-Hals Tumoren (2010) Landelijke richtlijn larynxcarcinoom, versie 3.0. http://www.oncoline.nl/larynxcarcinoom (Accessed Jan 2014)

[8] Nederlandse Werkgroep Hoofd-Hals Tumoren (2004) Landelijke richtlijn mondholte- en orofarynxcarcinoom, versie 1.4. http://www.oncoline.nl/mondholte-enorofarynxcarcinoom (Accessed Jan 2014)

[9] National Comprehensive Cancer Network (NCCN) (2013) NCCN clinical practice guidelines in oncology. Head Neck Cancer

[10] Gregoire V, Lefebvre JL, Licitra L, Felip E, EHNS-ESMO-ESTRO Guidelines Working Group (2010). Squamous cell carcinoma of the head and neck: EHNS-ESMO-ESTRO clinical practice guidelines for diagnosis, treatment and follow-up. Ann Oncol.

[11] Vermorken JB, Mesia R, Rivera F (2008). Platinum-based chemotherapy plus cetuximab in head and neck cancer. N Engl J Med.

[12] NVMO-commissie BOM (2009) Cetuximab in combinatie met platinabevattende chemotherapie bij inoperabel gerecidiveerd en/of gemetastaseerd plaveiselcelcarcinoom van het hoofd-hals gebied. http://www.nvmo.eu/files/_pdfs_bom/MO5%20%20nov%202009%20-%20Twee%20nieuwe%20adviezen%20commissie%20BOM.pdf (Accessed Jan 2014)

[13] Forastiere AA, Metch B, Schuller DE (1992). Randomized comparison of cisplatin plus fluorouracil and carboplatin plus fluorouracil versus methotrexate in advanced squamous-cell carcinoma of the head and neck: a Southwest Oncology Group study. J Clin Oncol.

[14] Nederlandse Werkgroep Hoofd-Halstumoren (2010) Hoofd-Hals Journaal 43. http://www.nwhht.nl/files/user/nr_43.pdf (Accessed Jan 2014)

[15] Singh B, Bhaya M, Stern J (1997). Validation of the Charlson comorbidity index in patients with head and neck cancer: a multi-institutional study. Laryngoscope.

[16] Quan H, Li B, Couris CM (2011). Updating and validating the Charlson comorbidity index and score for risk adjustment in hospital discharge abstracts using data from 6 countries. Am J Epidemiol.

[17] Tan SS, Hakkaart-van Roijen L, Al MJ (2008). A microcosting study of intensive care unit stay in the Netherlands. J Intensive Care Med.

[18] Tan SS, Van Gils CW, Franken MG, Hakkaart-van Roijen L, Uyl-de Groot CA (2010). The unit costs of inpatient hospital days, outpatient visits, and daycare treatments in the fields of oncology and hematology. Value Health.

[19] Merlano MC, Vermorken JB, Wilke H (2010). First-line treatment patterns for recurrent and/or metastatic head and neck cancer (R/M HNC) in Europe. J Clin Oncol.

[20] Chan ATC (2010). Nasopharyngeal carcinoma. Ann Oncol.

[21] De Mello RA, Gerós S, Alves MP, Moreira F, Avezedo I, Dinis J (2014). Cetuximab plus platinum-based chemotherapy in head and neck squamous cell carcinoma: a retrospective study in a single comprehensive European cancer institution. PLoS ONE.

[22] Chastek B, Harley C, Kallich J, Newcomer L, Paoli CJ, Teitelbaum AH (2012). Health care costs for patients with cancer at the end of life. J Oncol Pract.

[23] Uyl-deGroot CA, de Vries EGE, Verweij J, Sullivan R (2014). Dispelling the myths around cancer care delivery: it’s not all about costs. J Cancer Policy.

[24] Smith TJ, Hillner BE (2011). Bending the cost curve in cancer care. N Engl J Med.

[25] Earle CC, Landrum MB, Souza JM, Neville BA, Weeks JC, Ayanian JZ (2008). Aggressiveness of cancer care near the end of life: is it a quality-of-care issue?. J Clin Oncol.

[26] Greenhalgh J, Bagust A, Boland A (2009). Cetuximab for the treatment of recurrent and/or metastatic squamous cell carcinoma of the head and neck. Health Technol Assess.

[27] Hannouf MB, Sehgal C, Cao JQ, Mocanu JD, Winquist E, Zaric GS (2012). Cost-effectiveness of adding cetuximab to platinum-based chemotherapy for first-line treatment of recurrent or metastatic head and neck cancer. PLoS ONE.

